# Serum pigment epithelium-derived factor levels are independently correlated with the presence of coronary artery disease

**DOI:** 10.1186/1475-2840-12-56

**Published:** 2013-04-01

**Authors:** Feifei Wang, Xiaojing Ma, Mi Zhou, Xiaoping Pan, Jie Ni, Meifang Gao, Zhigang Lu, Jingyu Hang, Yuqian Bao, Weiping Jia

**Affiliations:** 1 Department of Endocrinology and Metabolism, Shanghai Jiao Tong University Affiliated Sixth People’s Hospital, Shanghai Clinical Center for Diabetes, Shanghai Key Clinical Center for Metabolic Disease, Shanghai Diabetes Institute, Shanghai Key Laboratory of Diabetes Mellitus, Shanghai, China; 2 Department of Cardiology, Shanghai Jiao Tong University Affiliated Sixth People’s Hospital, Shanghai, China

**Keywords:** Atherosclerosis, Coronary angiography, Coronary artery disease, Metabolic syndrome, Pigment epithelium-derived factor

## Abstract

**Background:**

Pigment epithelium-derived factor (PEDF) has been proved to be closely correlated with metabolic syndrome (MetS) and its components that are all risk factors of cardiovascular disease and may play a protective role against vascular injury and atherosclerosis. The present study was designed to investigate the relationship between serum PEDF and coronary artery disease (CAD).

**Methods:**

A total of 312 consecutive in-patients (including 228 with CAD and 197 with MetS) who underwent coronary angiography were enrolled. Serum PEDF was measured by sandwich enzyme immunoassay and used to carry out multivariate stepwise regression analysis to assess correlation with patient demographic and clinical parameters. Multiple logistic regression analysis was performed to identify factors independently correlated with CAD.

**Results:**

Patients with MetS had significantly higher levels of serum PEDF than non-MetS subjects (11.1(8.2, 14.2) vs. 10.1(7.6, 12.4) μg/mL; *P* < 0.05). Patients with CAD also had significantly higher serum PEDF than non-CAD subjects (11.0(8.1, 14.2) vs. 10.3(8.1, 12.8) μg/mL; *P* < 0.05). Triglyceride (TG), C-reactive protein (CRP), estimated glomerular filtration rate (eGFR), and hypoglycemic therapy were independently correlated with serum PEDF levels, and serum PEDF was independently positively correlated with CAD.

**Conclusions:**

Serum PEDF levels are independently positively associated with CAD in a Chinese population. Elevated PEDF may act as a protective response against vascular damage and subsequent CAD.

## Introduction

The pigment epithelium-derived factor (PEDF) belongs to the super family of serine protease inhibitors, and the major sources of circulating PEDF are liver and adipose tissues [1-3]. PEDF is characterized as a multifunctional protein possessing anti-angiogenic, anti-tumorigenic, anti-oxidant, anti-inflammatory, anti-thrombotic, and neuroprotective properties. Demonstrated as a highly effective anti-angiogenic factor, PEDF not only is capable of inhibiting vascular endothelial growth and migration, but can also suppress secretion of angiogenic factors [4,5] as well as activate the FAS-FAS ligand death pathway to stimulate endothelial cell apoptosis [6]. Nakamura et al. reported anti-oxidative effects of PEDF, showing that PEDF-mediated suppression of NADPH oxidase inhibited generation of reactive oxygen species and the subsequent neointimal hyperplasia induced by balloon injury [7]. Takenaka et al. also showed a cardio-protective function by which PEDF inhibited occlusive thrombus formation in the carotis artery of a rat model [8]. Finally, various studies have demonstrated the strong anti-inflammatory activities of PEDF; for example, PEDF was characterized both as a regulator of cytokines’ expression such as monocyte chemoattractant protein-1 and tumor necrosis factor-α as well as a mediator of macrophage and T cell function [9-11].

PEDF may rely on any of these protective properties to manifest a counteractive mechanism during the development of atherosclerosis and cardiovascular disease. Recent clinical findings have revealed that PEDF levels are closely associated with the presence of cardiovascular disease. Circulating PEDF levels were shown to be higher in subjects with metabolic syndrome (MetS) and to be correlated with the extent of MetS components [12-14]. The fact that MetS itself, and each of its components, are important risk factors of cardiovascular disease which has led researchers to hypothesize that increased PEDF levels might have occurred as a counter-regulatory response to the presence of vascular injury. A recent study showed that serum PEDF level was independently correlated with intima-media thickness and vascular inflammation, suggesting an association of PEDF with subclinical atherosclerosis in at least two aspects: morphological abnormalities of the vessel, and inflammation in the plaques [15].

The above clinical findings revealed that PEDF levels were closely associated with the presence of cardiovascular disease, however, no study to date has focused on the relationship between serum PEDF and coronary artery disease (CAD). Since such data may also help to identify PEDF as a promising therapeutic target for atherosclerosis and cardiovascular disease [16,17], the present study was carried out with Chinese patients who underwent coronary angiography to investigate the association between serum PEDF and CAD.

## Materials and methods

### Study population

A total of 312 participants (206 men and 106 women; age range: 38 ~ 86 years, mean age: 66.2 ± 10.1 years) who were admitted to the Department of Cardiology of Shanghai Jiao Tong University Affiliated Sixth People’s Hospital to undergo coronary angiography because they have once suffered or was just suffering from chest tightness and/or chest pain between July 2008 and January 2010 were enrolled in the study. Patients were excluded from enrollment according to: serious hepatic or renal dysfunction; acute myocardial infarction within the past three months; coronary by-pass surgery or percutaneous coronary intervention within the past six months; congestive heart failure (defined as New York Heart Association functional class III-IV); acute infection; or history of malignancy. All women were postmenopausal. All enrollees completed a standardized questionnaire to self-report past and present illnesses, medications, and smoking habits. Enrollees who identified themselves as regular smokers, or who reported smoking at least one cigarette per day for at least the past six months, were classified as current smokers [18]. The study was approved by the Ethics Committee of Shanghai Jiao Tong University affiliated Sixth People’s Hospital and complied with the Declaration of Helsinki. All the subjects provided written informed consent prior to the study participation.

According to the criteria of the 2007 Chinese Joint Committee for Developing Chinese Guidelines on Prevention and Treatment of Dyslipidemia in Adults [19], MetS was diagnosed if a patient had three or more of the following: 1) central obesity, defined as waist circumference (W) of >90 cm for men and >85 cm for women; 2) fasting triglyceride (TG) of ≥1.7 mmol/L, or receipt of specific treatment for previously diagnosed hypertriglyceridemia; 3) fasting high-density lipoprotein cholesterol (HDL-c) of <1.04 mmol/L, or receipt of specific treatment for previously diagnosed low HDL-c; 4) systolic blood pressure (SBP) of ≥130 mmHg and/or diastolic BP (DBP) of ≥85 mmHg, or receipt of treatment for previously diagnosed hypertension; 5) fasting plasma glucose (FPG) of ≥6.1 mmol/L and/or 2 h postprandial glucose (2hPG) of ≥7.8 mmol/L, or receipt of hypoglycemic therapy for previously diagnosed type 2 diabetes.

### Coronary angiography

Each study participant underwent coronary angiography by the standard Judkins technique [20]. All major coronary arteries were imaged in at least two orthogonal views. The angiographic analysis was performed by two experienced cardiologists who were blinded to the patients’ clinical information. Patients with ≥50% diameter lumen stenosis in a major coronary artery (left main coronary artery, left anterior descending artery or its first diagonal branch, left circumflex artery or its first obtuse marginal branch, and right coronary artery) were classified as CAD.

### Anthropometric evaluation

Each study participant underwent complete physical examination to obtain measurements of height, weight, W, and BP. Body mass index (BMI) was calculated as: [(weight in kg)/(height in m)^2^]. W was measured at the midpoint between the inferior margin of 12th rib and the iliac crest on the mid-axillary line.

### Laboratory measurements

Blood samples were collected after 10 h overnight fast and stored at −80°C until use. Study participants without a history of diabetes received the standard 75 g oral glucose tolerance test. All participants’ samples were assayed for FPG and 2hPG by the standard glucose oxidase method. Fasting insulin was measured via radioimmunoassay (Linco Research, St. Charles, MO, USA) and insulin resistance was estimated using the homeostasis model assessment index (HOMA-IR) [21]. Glycated hemoglobin (HbA1c) level was measured by high-pressure liquid chromatography (Bio-Rad Inc., Hercules, CA, USA). Serum creatinine (SCr), uric acid (UA), and lipid profiles, including TG, total cholesterol (TC), HDL-c and low-density lipoprotein cholesterol (LDL-c), were assayed by standard enzymatic procedures on an automated bioanalyzer (7600–020; Hitachi, Tokyo, Japan). The estimated glomerular filtration rate (eGFR; expressed as mL/min/1.73 m^2^) was calculated according to the equation from the Modification of Diet in Renal Disease (MDRD) study: [186 * (SCr/88.4)^-1.154^ * (age)^-0.203^ * 0.742 (if female)] [22]. Serum C-reactive protein (CRP) was measured by a particle-enhanced immunonephelometry assay (Dade Behring Inc., Newark, NJ, USA). The 24 h urine albumin (24hALB) concentration was determined by standard rate nephelometry method. Sandwich enzyme immunoassays were used to detect the levels of PEDF (BioVendor Laboratory Medicine, Modrice, Czech Republic) and adiponectin (Li Ka Shing, Faculty of Medicine, University of Hong Kong, China). The inter- and intra-assay coefficients of variation were <6.6% and <4.1% for PEDF, and <8.6% and <7.3% for adiponectin, respectively.

### Statistical analysis

All statistical analyses were carried out with the Statistical Package for Social Sciences software (version 16.0; SPSS, Chicago, IL, USA). The clinical and biochemical data of the subjects are presented as mean ± SD, except for skewed variables that are presented as median with interquartile range of 25-75%. Two-tailed tests and a 5% level of significance were applied for all statistical analyses. Intergroup comparisons of variables with normal distribution were carried out by the unpaired Student’s *t-*test; variables with non-normal distribution were compared by the Wilcoxon rank-sum test. For dichotomous or categorical variables, intergroup comparisons were carried out by the Chi-squared (χ^2^) test. Spearman’s correlation was used to assess the relation between serum PEDF and other clinical parameters. Multiple logistic regression analysis was performed to identify factors that were independently correlated with CAD; CAD was set as the dependent variable and age, BMI, PEDF, CRP, HOMA-IR, adiponectin, CAD family history, smoking status, hypoglycemic therapy, anti-hypertensive therapy, lipid-lowering therapy, components of MetS (including central obesity, hyperglycemia, hypertension, hypertriglyceridemia, and low HDL-c), as well as MetS itself were assessed as the independent variables. Multivariate stepwise regression analysis was used to further assess the independent correlated clinical parameters of serum PEDF; serum PEDF was set as the dependent variable and age, BMI, W, glucose levels, BP, lipid profiles, HOMA-IR, CRP, adiponectin, 24hALB, eGFR, UA, hypoglycemic, anti-hypertensive, and lipid-lowering therapy were assessed as the independent variables.

## Results

### Clinical characteristics of study participants

The overall average of serum PEDF was 10.9 (8.1, 13.8) μg/mL for the entire study population. The average serum PEDF level was not significantly different between the male and female patients (10.7 (8.0, 13.9) vs. 11.1 (8.4, 13.2) μg/mL; *P* > 0.05).

Compared with non-CAD subjects, CAD patients showed significantly higher age, 2hPG, HbA1c, and SCr, but significantly lower level of HDL-c (all *P* < 0.05; Table 1). The frequency of low HDL-c was significantly higher in CAD patients (*P* < 0.05), while the frequency of MetS and other components of MetS showed no difference between the CAD and non-CAD groups (all *P* > 0.05). In addition, the proportion of patients with hypoglycemic therapy and lipid-lowering therapy was significantly higher in the CAD group than in the non-CAD group (both *P* < 0.05).

**Table 1:** Characteristics of patients according to the presence or absence of CAD

**Parameters**	**Non-CAD**	**CAD**	***P***
n (male/female)	84 (43/41)	228 (163/65)	0.001
Age, years	64.0 ± 9.9	67.0 ± 10.0	0.018
Body mass index, kg/m^2^	24.8 ± 3.9	24.5 ± 3.0	0.520
Waist circumference, cm	89.6 ± 10.9	90.6 ±8.9	0.408
Fasting plasma glucose, mmol/L	5.5 (5.0, 6.0)	5.5 (5.0, 6.4)	0.448
2h postprandial glucose, mmol/L	8.2 (6.2, 9.8)	8.8 (6.7, 12.4)	0.031
Glycated hemoglobin A1c, %	6.0 (5.6, 6.5)	6.2 (5.8, 6.9)	0.005
Fasting insulin, mU/L	16.3(11.9, 22.2)	16.5 (12.0, 24.5)	0.431
HOMA-IR	4.0 (2.8, 6.0)	4.0(2.8, 5.9)	0.939
Systolic blood pressure, mmHg	130.0 (120.0, 150.0)	130 (120.0, 150.0)	0.335
Diastolic blood pressure, mmHg	80.0 (70.0, 85.8)	80.0 (70.0, 83.8)	0.569
Total cholesterol, mmol/L	4.5 ± 1.1	4.3 ± 1.1	0.070
Triglyceride, mmol/L	1.5 (1.0, 2.2)	1.5 (1.1, 2.2)	0.461
HDL-c, mmol/L	1.2 ± 0.3	1.1 ± 0.3	0.018
LDL-c, mmol/L	3.1 ± 0.9	2.9 ± 1.0	0.221
C-reactive protein, mg/L	1.4 (0.6, 3.6)	1.4 (0.6, 4.0)	0.329
Adiponectin, μg/mL	8.4 (5.6, 11,9)	7.1 (4.7, 12.1)	0.113
Serum creatinine, μmol/L	72.0 (61.0, 87.3)	79.0 (68.0, 91.0)	0.008
Uric acid, μmol/L	324.4 ± 85.5	352.3 ± 90.6	0.392
eGFR, mL/min/1.73m^2^	90.7 ± 25.1	85.5 ± 23.2	0.092
24h urine albumin, mg/d	7.5 (5.1, 14.1)	7.1 (4.9, 18.3)	0.775
CAD family history, n (%)	35 (41.6)	106 (46.5)	0.243
Smoking, n (%)	31 (36.9)	107 (46.9)	0.073
Metabolic syndrome, n (%)	51 (60.7)	146 (64.0)	0.340
Central obesity, n (%)	46 (56.1)	132 (58.9)	0.376
Hyperglycemia, n (%)	51 (60.7)	147 (64.5)	0.314
Hypertension, n (%)	71 (84.5)	206 (90.4)	0.109
Hypertriglyceridemia, n (%)	34 (40.5)	89 (39.0)	0.458
Low HDL-c, n (%)	33 (39.3)	117 (51.3)	0.039
Hypoglycemic therapy, n (%)	12 (14.3)	60 (26.3)	0.016
Anti-hypertensive therapy, n (%)	54 (64.3)	166 (72.8)	0.094
Lipid-lowering therapy, n (%)	11 (13.1)	80 (35.1)	<0.001

Data are mean ± SD or median (interquartile range). CAD, coronary artery disease; eGFR, estimated glomerular filtration rate; HDL-c, high-density lipoprotein cholesterol; HOMA-IR, homeostatic model assessment-insulin resistance; LDL-c, low-density lipoprotein cholesterol.

### Association of serum PEDF levels with MetS

**Figure 1:**
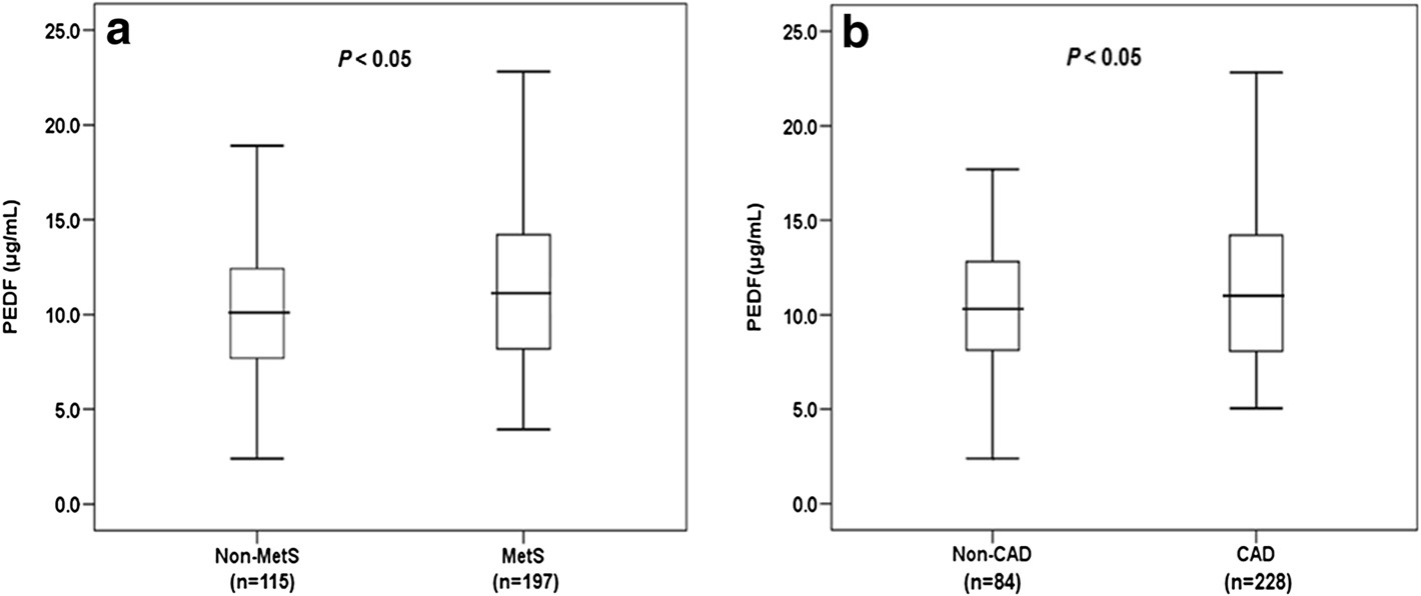
**Comparison of serum PEDF levels between study participants with and without MetS (a) and CAD (b).** The bars represent median, 25th and 75th percentile of PEDF level.

Serum PEDF levels were significantly higher in subjects with MetS than in those without MetS (11.1 (8.2, 14.2) vs. 10.1(7.6, 12.4) μg/mL; *P* < 0.05; Figure 1a). Spearman’s correlation analysis showed that serum PEDF was positively correlated with hypertriglyceridemia (R = 0.213, *P* < 0.001), but no significant correlation was observed among other components of MetS.

### Correlation of serum PEDF and CAD

As shown in Figure 1b, CAD patients had significantly higher levels of serum PEDF than non-CAD subjects (11.0 (8.1, 14.2) vs. 10.3 (8.1, 12.8) μg/mL; *P* < 0.05). Multivariate logistic regression analysis indicated that age, PEDF and lipid-lowering therapy were independently positively correlated with CAD (Table 2).

**Table 2:** Multivariate logistic regression analysis showing factors independently associated with CAD

**Independent variable**	**B**	**S.E.**	***P***	**OR**	**95% CI**
Age	0.028	0.014	0.037	1.029	1.002-1.057
PEDF^a^	1.661	0.773	0.022	5.267	1.157-23.978
Lipid-lowering therapy	1.076	0.360	0.003	2.933	1.449-5.934

Variables of the original model included: age, BMI, PEDF, CRP, HOMA-IR, adiponectin, central obesity, hyperglycemia, hypertension, hypertriglyceridemia, low HDL-c, MetS, CAD family history, smoking, hypoglycemic therapy, anti-hypertensive therapy, and lipid-lowering therapy.

^a^ Logarithmically transformed before analysis.

### Influencing factors of serum PEDF levels

Table 3 shows the correlation analysis results for serum PEDF with the various anthropometric variables and clinical parameters. Serum PEDF was found to be positively correlated with FPG, HbA1c, HOMA-IR, TG, CRP, SCr and UA, and negatively correlated with eGFR. Stepwise multivariate regression analysis indicated that TG, CRP, eGFR, and hypoglycemic therapy were independently correlated with serum PEDF (Table 4).

**Table 3:** Correlation between clinical characteristics and serum PEDF

**Variable**	**R**	***P***
Age	0.024	0.673
Gender	0.015	0.794
Body mass index	0.062	0.277
Waist circumference	0.095	0.096
Fasting plasma glucose	0.138	0.015
2h postprandial glucose	0.078	0.167
Glycated hemoglobin A1c	0.114	0.047
HOMA-IR	0.139	0.019
Systolic blood pressure	0.040	0.480
Diastolic blood pressure	−0.048	0.397
Total cholesterol	0.093	0.104
Triglyceride	0.263	<0.001
High-density lipoprotein cholesterol	−0.052	0.362
Low-density lipoprotein cholesterol	0.005	0.932
C-reactive protein	0.191	0.001
Adiponectin	−0.032	0.577
Serum creatinine	0.187	0.001
Estimated glomerular filtration rate	−0.203	<0.001
Uric acid	0.152	0.008
24h urine albumin	0.120	0.052

**Table 4:** Stepwise multivariate regression analysis of serum PEDF levels

**Independent variable**	**Β**	**S.E.**	**Standardized β**	***P***
Triglyceride ^a^	0.167	0.046	0.239	<0.001
CRP ^a^	0.044	0.019	0.148	0.022
eGFR	−0.001	0.000	−0.173	0.008
Hypoglycemic therapy	0.085	0.028	0.191	0.003

Variables of the original model included: age, BMI, W, FPG, 2hPG, HbA1c, SBP, DBP, TC, TG, HDL-c, LDL-c, HOMA-IR, CRP, adiponectin, 24hALB, eGFR, UA, hypoglycemic therapy, anti-hypertensive therapy, and lipid-lowering therapy.

^a^ Logarithmically transformed before analysis.

## Discussion

MetS is well recognized as an aggregate of cardio-metabolic risk factors subsequent cardiovascular disease and type 2 diabetes mellitus [23,24], and the association of PEDF with MetS and its components has been verified in previous studies [12-14]. In a 10-year prospective study, enhanced PEDF level was identified as an independent predictor for the development of MetS in men [13], which further affirmed the prediction effect of PEDF on MetS. However, these studies focused solely on subjects with CAD risk factors but no clinical evidence of cardiovascular disease, which may have limited their ability to extend this relation of serum PEDF with MetS to the subsequent onset of cardiovascular disease. To overcome this limitation, the present study enrolled CAD and non-CAD subjects (~3:4 ratio) as well as MetS and non-MetS subjects (~2:3 ratio) and still found that serum PEDF level was significantly increased in the MetS patient group. This finding agrees with previous functional studies that indicated enhanced levels of PEDF might exert a counteractive activity against simultaneous enhancement of CAD risk factors [13,14].

In the current study of the broad panel of MetS pathogenic components, PEDF was found to be significantly positively correlated with hypertriglyceridemia, and TG was identified as an independent influencing factor of serum PEDF levels. Dyslipidemia is known to play an important role in the development of atherosclerosis and CAD. Specifically, long-term dyslipidemia has been shown to cause physical injury to the vascular intima, and the accompanying pro-inflammatory state of the tissue promotes atherosclerosis and CAD. In clinical studies, patients with elevated TG levels developed CAD more frequently than their counterparts with TG levels in the normal range and TG has been identified as an independent risk factor for coronary events [25,26]. In the current study, TG was found to be strongly and independently correlated with serum PEDF in patients with CAD or at high risk of CAD. Moreover, these findings also agree with the previous studies in patients without CAD history [12,13]. It is possible that the enhanced levels of serum PEDF in CAD or CAD-risk patients reflect a response to hypertriglyceridemia, whereby the body is attempting to correct the perturbance in the lipid metabolism system [27]. Intriguingly, we failed to find any significant association of serum PEDF with the other two principal components of MetS, central obesity and hypertension. These negative findings may be explained by the fact that a large portion of our subjects had been receiving therapeutic treatments for hyperglycemia, hypertension, or hyperlipidemia for long periods prior to study participation; to account for this potential confounding aspect, hypoglycemia therapy was entered the equation in the multiple regression analysis of PEDF levels. Body weight and W in subjects of this study might have changed in response to a long-term influence of medications; therefore, the relation of PEDF with obesity might be masked by other determinants.

Substantial evidence exists to support the hypothesis that PEDF plays a protective role against microangiopathy; moreover, PEDF has been proposed as a potential protective factor against diabetic microvascular complications [28-30]. Yet, few studies to date have investigated the association of PEDF with macroangiopathy, as was done in the current study which demonstrated for the first time that serum PEDF was independently positively correlated with CAD in a Chinese population. This finding contradicts a previous study by Shiga et al., which showed no significant correlation between PEDF and the presence of CAD in a Japanese population [31]; however, this apparent inconsistency may merely indicate the different inclusion subjects.

Atherosclerosis, which is considered the pathological basis of CAD, is a chronic inflammatory disorder [32]. Previous studies have shown that CRP was positively correlated with PEDF, and suggested that this relation may reflect the body’s attempt to suppress a detrimental inflammatory reaction involving the endothelial cells [14,33]. In our study, we found no difference in the CRP levels of CAD and non-CAD patients, possibly because the majority of those non-CAD patients actually possessed many of the CAD risk factors and >60% of them were MetS patients. Regardless, the multivariate stepwise regression analysis performed for the study population identified CRP as an independent influencing factor of serum PEDF levels, and this finding agrees with the proposed association of PEDF with chronic inflammation. Furthermore, oxidative stress, which is elevated in patients with metabolic disorder or CAD, could be one of the triggers in the liver (an important source organ of circulating PEDF), as hydrogen peroxide has been shown to induce PEDF expression in human hepatocytes [34].

Given the fact that PEDF exerts anti-angiogenic, anti-oxidative, anti-thrombotic, and anti-inflammatory properties on vascular tissues, researchers have speculated that PEDF levels might be elevated to counteract generation of a pro-atherosclerotic environment induced by vascular injuries [16,17]. Ueda et al. reported that PEDF injection suppressed cardiac fibrosis, inhibited tissue remodeling and improved cardiac function in a rat model of acute myocardial infarction and suggested that PEDF may be a novel therapeutic strategy for human acute myocardial infarction [35]. Since PEDF exerts a number of protective effects on vascular and myocardial tissues, elevated serum PEDF levels in CAD patients may also play a counter-regulatory and protective role against vascular damage caused by hypertriglyceridemia, hyperglycemia, and chronic inflammation.

Moreover, Rychli et al. found that PEDF was significantly associated with CAD (for trend, *P* = 0.037) and correlated with rehospitalization for heart failure (HF) worsening, with a more prominent risk increase association in CAD patients; these previous findings further support our current results. Rychli et al. also suggested that PEDF was associated with chronic deterioration of cardiomyopathy and played a role in the progression of HF by inducing apoptosis of human cardiac myocytes and fibroblasts [36]. We believe that the elevated PEDF observed in CAD patients occurs in response to vascular injuries, chronic inflammation, and oxidative stress, and that its function involves preventing CAD deterioration.

It has been reported that cardiovascular disease was closely regulated through the signaling pathways of the mammalian target of rapamycin (mTOR) which was associated with endothelial cell survival and growth, as well as cardiomyocyte proliferation [37]. Wang et al. confirmed the antiangiogenic property of PEDF and firstly reported that insulin could down-regulate PEDF expression, which at least partly depended on mTOR kinase as the inhibitory effect of insulin on PEDF expression would disappear when added the mTOR inhibitor rapamycin [38]. It is possible that PEDF would offer exciting prospects for the development of new therapies for cardiovascular disease.

Another physiological factor that was correlated with the elevated PEDF levels in our study population was eGFR (independently negatively correlated). This finding is not surprising since renal filtration is known to affect serum PEDF levels, and is in line with findings from previous studies [15,31].

## Limitations

When interpreting the results from our present study, it is important to consider some of the features of the study design that may limit the generalizability of our findings. Firstly, the cross-sectional design of the current study precluded our ability to determine the cause-effect relationship for PEDF and CAD. Secondly, the study population was relatively small, and larger prospective studies are necessary to confirm the role of PEDF in the development of CAD. Moreover, the study population was composed of individuals who were suspected of CAD (admitted to hospital to undergo coronary angiography) and most had several of the known risk factors of CAD, which may have biased the results.

## Conclusions

Nonetheless, the population of Chinese patients assessed in this study demonstrated a significantly independent correlation of serum PEDF with the presence of CAD. Biologically, PEDF is capable of exerting a number of protective effects under conditions of metabolic disorder, and the mechanism fits with the observation of elevated serum PEDF in patients with MetS and CAD. In particular, elevated serum PEDF levels may represent a counter-regulatory mechanism that acts as a protective response against vascular damage and subsequent CAD. Molecular targeting of PEDF might be a promising therapeutic strategy for treatment of cardiometabolic disorders and represent a useful predictive index for treatment effectiveness. Further laboratory and clinical studies are warranted to confirm the protective effect of PEDF on cardiovascular disease and to assess its potential as a therapeutic target.

## Abbreviations

BMI: Body mass index; BP: Blood pressure; CAD: Coronary artery disease; CRP: C-reactive protein; eGFR: Estimated glomerular filtration rate; FPG: Fasting plasma glucose; HbA1c: Glycated hemoglobin A1c; HDL-c: High-density lipoprotein cholesterol; HOMA-IR: Homeostasis model assessment-insulin resistance; LDL-c: Low-density lipoprotein cholesterol; MetS: Metabolic syndrome; PEDF: Pigment epithelium-derived factor; SCr: Serum creatinine; TC: Total cholesterol; TG: Triglyceride; UA: Uric acid; W: Waist circumference; 2hPG: 2 h postprandial glucose; 24hALB: 24 h urine albumin.

## Competing interests

All authors declare that they have no competing interests.

## Authors’ contributions

YB and WJ designed the study. MZ, JN, and MG collected the data. FW analyzed the data and drafted the manuscript. XP performed the PEDF measurements. ZL and JH carried out the angiographic analysis. XM, YB, and WJ revised the manuscript and contributed to discussion. All authors read and approved the final manuscript.
